# Reduced myopia progression with low-dose atropine in DIMS non-responders and evidence of altered pupillary light reflex kinetics

**DOI:** 10.1038/s41598-026-50070-8

**Published:** 2026-07-13

**Authors:** Patricia Domsa, Attila Törcsvári, Viktória Baross, Judit Körtvélyes, Rita Széchey, Adrienne Csutak, Éva M Bankó

**Affiliations:** 1 Non Plus Ultra Vision Centre, Budapest, Hungary; 2 Department of Ophthalmology, University of Pécs Medical School, Pécs, Hungary; 3 Arcanum Development Ltd, Budapest, Hungary; 4 Semmelweis University of Medicine, Budapest, Hungary; 5 Heim Pál National Paediatric Institute, Budapest, Hungary; 6 HUN-REN Research Centre for Natural Sciences, Magyar tudósok krt. 2, Budapest, 1117 Hungary

**Keywords:** Myopia, Defocus incorporated multiple segments (DIMS), Atropine, Dynamic pupillometry, Pupil constriction, Diseases, Health care, Medical research

## Abstract

This study evaluated the efficacy of adding nightly 0.025% atropine to Defocus Incorporated Multiple Segments (DIMS) spectacle treatment in children with ongoing myopia progression and explored whether dynamic pupillometry could help identify such progression. In a mixed-method design, a longitudinal arm included 20 DIMS non-responders, defined by annual progression of ≥ 0.5 D spherical equivalent refraction or ≥ 0.2 mm axial elongation, who received adjunctive atropine and were followed for 12 months. In a separate cross-sectional arm, dynamic pupillometry was performed in 60 DIMS-treated children classified as fast or slow progressors based on prior-year progression. Combination therapy significantly reduced myopia progression, with mean axial elongation decreasing from 0.39 ± 0.18 to 0.20 ± 0.18 mm/year and mean refractive progression improving from − 0.82 ± 0.67 to − 0.28 ± 0.45 D/year. Transient axial shortening was observed in 20% of patients, while 25% remained unresponsive, predominantly those with high maternal myopia. Dynamic pupillometry revealed that fast progressors exhibited slower and prolonged pupil constriction compared with slow progressors, whereas dilation parameters and static pupil size did not differ. In conclusion, adjunctive low-dose atropine effectively reduced progression in most DIMS non-responders, and altered pupil constriction dynamics may reflect underlying physiological differences associated with treatment resistance.

## Introduction

Myopia has become a major global public health concern, with prevalence rising sharply over recent decades. In Europe, national screening programs report rates of 20–30% in most countries, reaching 40–50% among teenagers and young adults in some nations^1,2^. This surge underscores the urgent need for effective intervention protocols to control progression and prevent long-term complications such as myopic maculopathy, retinal detachment, and glaucoma. Among current optical interventions, Defocus Incorporated Multiple Segments (DIMS) spectacle lenses have emerged as a leading non-invasive option^3^, with proven efficacy across diverse populations including Europe^4–6^. However, treatment success is not universal: younger age, astigmatism, and high maternal myopia have been identified as risk factors for suboptimal response^6^.

For children demonstrating continued progression despite DIMS therapy, combination approaches are warranted. Adding low-dose atropine enhances efficacy beyond optical treatment alone^4,7^, with 0.025% offering a favourable balance between effect and tolerability in European populations^7,8^. Yet clinicians still lack reliable tools to predict who will become a DIMS non-responder or when escalation should occur. Identifying physiological biomarkers that reveal early or ongoing progression could enable more timely, individualized intervention.

Both atropine and DIMS lenses act, at least in part, through mechanisms involving retinal signalling pathways and modulation of choroidal structure and perfusion^9–12^. Choroidal circulation and thickness are under autonomic control, and autonomic tone can influence retinal neurotransmitter activity. The pupil, in turn, is governed exclusively by distinct sympathetic and parasympathetic pathways^13,14^, making its dynamics an accessible, non-invasive reflection of underlying autonomic processes. This link has generated growing interest in dynamic pupillometry as a potential real-time indicator of myopia-related physiological states^15–18^. However, the relationship between pupillary light reflex characteristics and myopia progression remains underexplored^19^, particularly in children already undergoing optical treatment.

In this context, we first introduced low-dose (0.025%) atropine as adjunct therapy in DIMS non-responders (defined as ≥ 0.5 D spherical equivalent refraction (SER) increase or ≥ 0.2 mm axial elongation/year) and compared axial length and refractive changes before and after combination therapy to evaluate whether progression could be halted. Building on this interventional cohort, we subsequently examined whether dynamic pupillometry could distinguish fast progressors from slow progressors. Dynamic pupillometry parameters—such as constriction velocity, amplitude, latency, and recovery dynamics—were recorded in DIMS-treated children grouped according to their recent rate of progression. Age- and refractive-status matching allowed us to isolate physiological differences related specifically to treatment response, as both age and refractive status had been shown to independently influence pupil responses^20,21^.

By combining therapeutic outcome tracking with functional physiological analysis, our study focuses on a critical and understudied subgroup—children who continue to progress despite evidence-based optical therapy. We propose that incorporating objective measures such as dynamic pupillometry into clinical evaluation may support more timely and individualized escalation of myopia control strategies, helping to shift management from reactive to predictive.

## Materials and methods

### Study design

The study employed a mixed-methods design, combining interventional and observational components, with data collected in a paediatric ophthalmology private practice in Budapest. The interventional longitudinal arm included DIMS non-responders—patients showing significant myopia progression during the previous year despite DIMS lens use—who received adjunct nightly 0.025% atropine and were monitored for 6 and 12 months to evaluate treatment response. The retrospective cross-sectional arm assessed dynamic pupillometry in myopic children grouped by their recent progression rate under DIMS therapy. Fast progressors were age- and myopia-matched with slow or non-progressors. Key outcomes included spherical equivalent refraction (SER) and axial length (AL) in the longitudinal study, measured at baseline, 6, and 12 months, and pupillometric parameters (pupil diameter and constriction/dilation velocities) in the cross-sectional analysis. Comparisons between combination therapy and prior DIMS monotherapy were based on AL and SER changes from the preceding 6- and 12-month intervals. The study was approved by the National Centre for Public Health and Pharmacy of Hungary and adhered to the tenets of the Declaration of Helsinki.

### Participants

The study included altogether 72 ethnic Caucasian participants aged 6–17 years (mean ± SD: 11.0 ± 2.5 years; 39 females). Informed consent was obtained from all the participants and/or their legal guardians. For detailed demographics of each arm, refer to Table 1. Myopia was defined as SER ≤–0.5 D. Progression during DIMS therapy was classified as fast when annual change reached ≥ 0.5 D in SER or ≥ 0.2 mm in axial length, consistent with commonly used clinical threshold for progressive myopia and with IMI guidance^22^ indicating that axial elongation of 0.2–0.3 mm/year is associated with increasing myopia. On the other hand, slow or non-progression was classified as values below these thresholds. In DIMS non-responders with clinical indicators of further progression (age, dioptric status, family history), adjunct 0.025% atropine therapy was initiated following guardian consent. Exclusion criteria included ocular pathology other than myopia and refractive errors exceeding − 10.0 SPH or + 4.0 CYL, reflecting early DIMS lens limitations.

Combination therapy was started in 22 DIMS non-responders, with 20 completing follow-up and included in analysis. The cross-sectional study initially enrolled 37 fast progressors (including the 22 in combination therapy) and 37 age- and dioptre-matched slow progressors. After exclusion for poor pupillometry quality (e.g., eyelid closure during light stimulus), 26 fast and 34 slow progressors remained. Eight patients overlapped between the longitudinal and cross-sectional cohorts. Optical and demographic characteristics are summarized in Table 1.

**Table 1:** Patient baseline characteristics for each group. Values indicate mean ± SD and [min – max].

	Combined treatment	DIMS non-responders	DIMS responders (control)
	*N* = 20	*N* = 26	*N* = 34
Age at enrolment (*years*)	9.9 ± 2.4 [5–16]	10.4 ± 2.1 [7–16]	11.8 ± 2.5 [7–17]
*Gender*			
Male, % (n)	40% (8)	35% (9)	53% (18)
Female, % (n)	60% (12)	65% (17)	47% (16)
*Cycloplegic autorefraction in SER (D)*			
Right eye	– 6.1 ± 2.5 [– 9.88 – – 1]	– 4.9 ± 2.4 [– 13 – – 1.88]	– 3.9 ± 2.2 [– 10.25 – – 0.75]
Left eye	– 5.9 ± 2.3 [– 9.88 – – 0.75]	– 4.9 ± 2.5 [– 14 – – 2.13]	– 3.7 ± 2.0 [– 10.88 – – 0.5]
*Axial length (mm)*			
Right eye	25.23 ± 1.00 [23.63–27.15]	24.76 ± 1.29 [23.14–29.72]	24.61 ± 1.09 [22.93–27.44]
Left eye	25.07 ± 0.99 [23.05–26.72]	24.73 ± 1.31 [22.96–29.83]	24.54 ± 0.97 [23.0–27.46]
DIMS therapy duration (*years*)	1.5 ± 0.8 [1–2.6]	1.5 ± 0.7 [1–2.7]	1.3 ± 0.6 [1–2.7]

SER D: spherical equivalent refraction dioptre.

### Intervention

All participants had been previously prescribed DIMS spectacles (Hoya MiyoSmart^®^, Tokyo, Japan) and were instructed to wear them during waking hours replacing their single vision spectacles. Participants in the longitudinal study were additionally given compounded low-dose atropine (0.025%) instructed to instill one drop in each eye every evening and were informed of the potential side effects. Adherence to DIMS spectacle wear and nightly atropine use was assessed at each follow-up visit by asking the child and parent about treatment wear, tolerance, and any difficulties with the prescribed regimen.

### Procedures

Clinical measurements included best corrected visual acuity, autorefraction (Topcon KR-800, Tokyo, Japan) conducted in cycloplegia (achieved through two drops of 10 mg/ml (1%) cyclopentolate (Laboratório Edol Produtos Farmacéuticos, S.A., Portugal), with the second administered at 15 min and refractometry conducted 25 min after the second drop), as well as AL measurements (Topcon Myah, Topcon Healthcare, Tokyo, Japan). Furthermore, a dilated fundoscopy was performed for every patient (SM-70 N, Takagi Seico Co. Ltd., Tokyo, Japan) with a Volk digital wide field lens (Volk Optical, Mentor, OH, USA).

Dynamic pupillometry was performed using the Topcon Myah system (Topcon Healthcare, Tokyo, Japan) with its dedicated pupillometry module to record the pupillary light reflex. All dynamic pupillometry measurements were conducted before cycloplegia and the pupillometry measurement analysed was taken at baseline, i.e. when the potential decision to treat with adjunct atropine was made. Following 5 min of dark adaptation, three consecutive measurements were obtained for each eye, starting with the right. Each acquisition consisted of 3000 ms under scotopic conditions, a 500-ms light flash (1.1 cd/m² at source), and a subsequent 9000-ms dark recovery period. Data were exported via i-Map Pro software (Visia Imaging, San Giovanni Valdarno, Italy; Topcon Corporation subsidiary) and processed in MATLAB (MathWorks Inc., Natick, MA, USA).

Scotopic and photopic pupil diameters were defined as the maximal diameter within the 100-ms window preceding the light stimulus and the minimal diameter following stimulus onset, respectively. Absolute constriction amplitude (ACA) was calculated as their difference. The constriction phase was characterized by maximum and mean constriction velocities, the latter determined between stimulus onset and the point of minimal diameter. The dilation phase was described by the maximum dilation velocity during the 2000-ms period following maximal constriction. Corresponding timepoints for each diameter parameter were also analysed.

### Statistical analysis

Descriptive statistics are presented as means ± standard deviation. Data from both eyes were analysed within the same mixed-effect model using “eye” as a random factor, except for dynamic pupillometry, where only right-eye data were included because left-eye responses could be influenced by prior light exposure to the right eye. Normality and homogeneity of variance were verified using one-sample Kolmogorov–Smirnov and Levene tests, respectively. In the interventional study, because the timing of follow-up visits was not identical for all participants, outcomes were adjusted for the actual elapsed time between measurements. The yearly rate of progression was derived from the slope of the best-fitting regression line for each participant, separately for the pre- and post-atropine periods. In addition, changes in AL and SER at the twelve month visit before and following low-dose atropine were analysed similary using longitudinal mixed-effects models : change-from-baseline values before (baseline – pre) and after (post – baseline) atropine introduction were used as dependent variables, with subject and eye as random effects, and “therapy” (before vs. after atropine) as a fixed effect, with their respective interaction terms. In addition, in the cross-sectional analysis, age, refractive error, and duration of DIMS wear were compared between fast and slow progressing groups with two-sample Student’s *t*-tests. Pupillometry outcomes were evaluated using ANCOVA models with “group” as a between-subject factor and “age,” “baseline myopia (SER),” and “DIMS duration” as covariates. To control for multiple comparisons, a Bonferroni-adjusted significance thresholds of *p* < 0.012 and *p* < 0.025 were used for the four variables related to constriction and the two variables related to dilation, respectively. All analyses were performed in Statistica 14 (TIBCO Software Inc., Palo Alto, CA, USA).

## Results

### Combined DIMS—atropine therapy

**Fig. 1:**
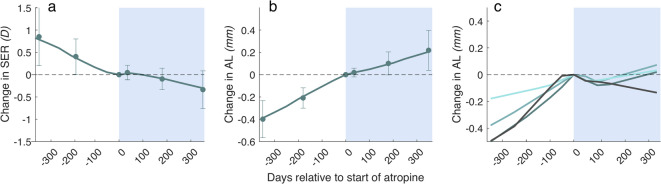
Effect of atropine on myopia progression using DIMS lenses. SER (**a**) and AL (**b**) change relative to baseline, i.e. the start of adjuvant atropine therapy. Light grey shaded area indicates combination therapy, while the white area corresponds to DIMS therapy alone. Datapoints represent gathered data, while the continuous line represents interpolated data used for calculating the rate of change. Error bars represent Std; *N* = 20. (**c**) AL change of those individuals, who had shown a decrease as a result of combination therapy, most likely as a result of transient choroidal thickening.

Among DIMS non-responders who completed adjunct 0.025% atropine therapy (*N* = 20), myopia progression significantly declined compared with their prior DIMS-only period (Fig. 1a-b, Table 2a.). The rate of mean axial elongation decreased from 0.39 ± 0.18 mm/year to 0.20 ± 0.18 mm/year (therapy main effect: F_(1,19)_ = 13.96, *p* = 0.0032), while SER change improved from − 0.82 ± 0.67 D/year to − 0.28 ± 0.45 D/year (F_(1,19)_ = 9.72, *p* = 0.020). Similarly, the change in AL and SER during the 12 months preceding atropine initiation exceeded that observed in the first 12 months of combination therapy (F_(1,19)_ = 15.71, *p* = 0.0017 for AL; F_(1,19)_ = 9.97, *p* = 0.015 for SER). There were no main effect or interaction of ‘eye’ in any of the analyses, however, there were significant subject x therapy interaction in all the models ( all Fs ≥ 4.6, all ps ≤ 0.0008) indicating that therapy effect was not homogenous across patients (Table 2b-c). Indeed, a subset of four patients showed transient axial shortening following atropine administration, consistent with choroidal thickening (Fig. 1c). In contrast, five patients remained unresponsive to combination therapy; notably, four of these had severe maternal (≤–10 D) and moderate paternal (≤–4 D) myopia, suggesting a genetic contribution to treatment resistance.

**Table 2:** Change in axial length (AL) and spherical equivalent (SER) relative to the start of atropine administration (a) in the whole group; (b) change in AL in the subgroup where AL shortening was observed, and (c) in the subgroup where combination therapy was not affective. ATR: atropine; data indicate mean ± std.

	Eye	12 m before ATR	6 m before ATR	6 m after ATR	12 m after ATR
a	(N = 20)				
AL	R	− 0.40 ± 0.16 mm	− 0.21 ± 0.09 mm	0.12 ± 0.10 mm	0.23 ± 0.19 mm
	L	− 0.39 ± 0.19 mm	− 0.21 ± 0.10 mm	0.09 ± 0.12 mm	0.19 ± 0.19 mm
SER	R	0.83 ± 0.55 D	0.44 ± 0.41 D	− 0.03 ± 0.26 D	− 0.23 ± 0.44 D
	L	0.76 ± 0.69 D	0.38 ± 0.50 D	− 0.07 ± 0.32 D	− 0.34 ± 0.47 D
b	(N = 4)				
AL	R	− 0.36 ± 0.16 mm	− 0.21 ± 0.09 mm	0.02 ± 0.06 mm	0.08 ± 0.09 mm
	L	− 0.45 ± 0.18 mm	− 0.25 ± 0.08 mm	0.10 ± 0.06 mm	0.08 ± 0.11 mm
c	(N = 5)				
AL	R	− 0.36 ± 0.12 mm	− 0.18 ± 0.06 mm	0.21 ± 0.04 mm	0.39 ± 0.08 mm
	L	− 0.34 ± 0.14 mm	− 0.16 ± 0.09 mm	0.20 ± 0.05 mm	0.39 ± 0.04 mm

### Pupillometry marker for progression

Pupillometry parameters were compared between fast progressors despite DIMS therapy (≥ 0.5 D SER or ≥ 0.2 mm AL/year) and age- and dioptre-matched slow progressors (< 0.5 D, < 0.2 mm/year) to identify potential physiological markers of progression. After excluding recordings of poor quality (blinks during stimulus), analysed fast progressors (*N* = 26) were slightly younger and more myopic than slow progressors (*N* = 34) (Table 1; t_(60)_=–2.32, *p* = 0.024 and t_(60)_=–1.76, *p* = 0.083, respectively), whereas DIMS treatment duration did not differ significantly between groups (≈ 1.5 years; t_(60)_ = 1.12, *p* = 0.27). These variables were included as covariates in subsequent analyses.

**Fig. 2:**
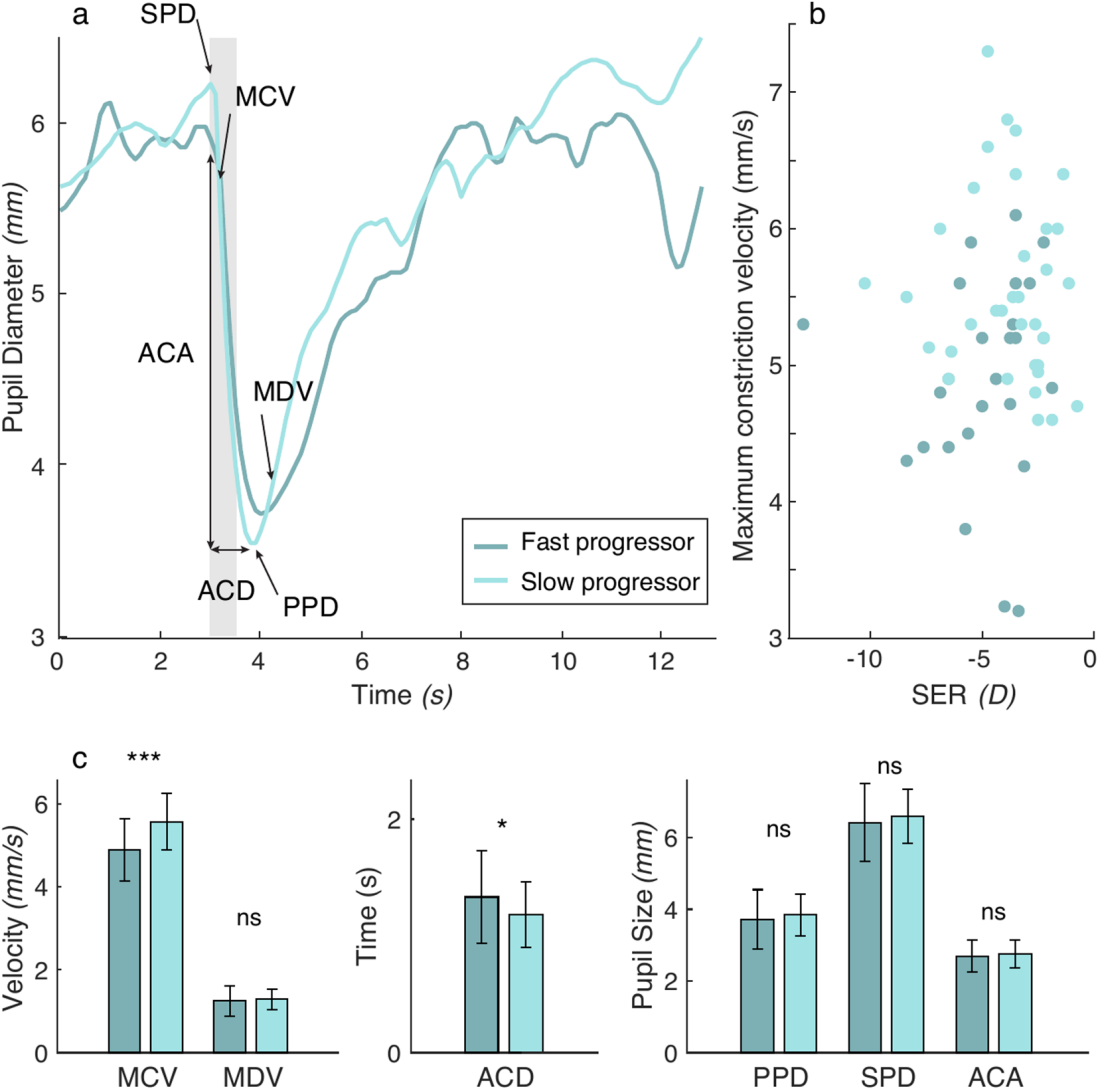
Pupillary Light Reflex. (**a**) Dynamic pupillometry curves of a typical pair of fast progressor (dark) and slow progressor (light) patient. Grey shaded area indicates illumination. (**b**) Maximum constriction velocity values for all participating patients as a function of their spherical equivalent refraction. Data show decreased velocity values for fast-progressors, while refraction has no effect on velocity. (**c**) Mean and standard deviation of each analysed measure: maximum constriction and dilation velocity (MCV and MDV, respectively); absolute constriction duration (ACD); and photopic, scotopic pupil diameter (PPD and SPD, respectively), and absolute constriction amplitude (ACA). ***p < 0.001, *0.012 < *p* < 0.05, ns: non-significant. N_fast progressor_ = 26, N_slow progressor_ = 34.

Fast progressors exhibited a consistently slower and more prolonged constriction phase of the pupillary light reflex compared with slow progressors (Fig. 2). Both maximum and average constriction velocities were significantly reduced (main effect of group: F_(1,55)_ = 14.57, *p* = 0.0003; F_(1,55)_ = 11.40, *p* = 0.0014), while there was a strong tendency for constriction duration being longer (F_(1,55)_ = 4.55, *p* = 0.037) and time to peak velocity delayed (F_(1,55)_ = 4.02, *p* = 0.05), which did not reach the Bonferroni corrected significance threshold (*p* < 0.012). In contrast, dilation-phase parameters (maximum velocity and latency; all Fs ≤1.26, *ps*≥ 0.27) and pupil diameters under various lighting conditions (photopic, scotopic, and absolute constriction amplitude; all Fs ≤1.37, *ps*≥ 0.25) did not differ between groups. Covariates (age, DIMS duration, baseline dioptre) showed no systematic effects.

## Discussion

In this study, we first demonstrated that introducing low-dose (0.025%) atropine as adjunct therapy effectively reduced both axial elongation and refractive progression in children who continued to progress despite DIMS spectacle wear. This finding underscores the value of early treatment escalation in DIMS non-responders. Building on these clinical results, we explored whether dynamic pupillometry could help identify children at risk of such treatment resistance. Progressing patients exhibited significantly slower and prolonged pupil constriction compared with stable responders, while dilation dynamics and baseline pupil diameters showed no differences. Together, these findings suggest that dynamic pupillometry reflects physiological variations linked to treatment response and could, with improved sensitivity or related measures, evolve into a clinically useful biomarker for guiding individualized myopia control.

### Combination therapy: atropine and DIMS

Low-dose atropine has been shown to reduce myopia progression in a dose-dependent manner across multiple randomized clinical trials, with concentrations between 0.01% and 0.05% balancing efficacy and side effects^7,8,23^. Our study supports the growing evidence of the synergistic benefits of the combination of myopia control spectacles – including DIMS– plus atropine therapy^4,7,24,25^ and longitudinally extend it by showing that combining optical and pharmacological approaches can achieve superior control in patients insufficiently managed by monotherapy. To the best of our knowledge, the only longitudinal study similar in approach was a recent study by Sim et al.^26^, where they show that the addition of HALT lenses can stop myopia progression in the majority of patients resistant to low-dose atropine monotherapy. In contrast, our study focused on a European population and targeted children who continued to progress despite evidence-based optical treatment with DIMS lenses, introducing 0.025% atropine as an adjunct therapy. The fact that our results parallel those of Sim et al. strengthens the evidence that optical and pharmacological approaches can be flexibly combined across different populations, supporting the broader applicability of combination strategies in myopia control. Importantly, we observed tolerability of 0.025% atropine without major side effects, in line with recent reports^8,23,26^.

A subset of patients (20%) showed transient decreases in MYAH-derived axial length following the introduction of low-dose atropine. This finding mirrors that of Sim et al.^26^, who reported short-term hyperopic axial shifts in 24% of treated children. As previous studies have documented choroidal thickening with both low-dose atropine^9,27^ and myopic-defocus optics^10,28^, these small axial-length reductions likely reflect transient choroidal expansion, although choroidal thickness was not directly measured in our study. This observation supports the notion that atropine may exert part of its myopia-control effect through modulation of the choroid^11^. While not observed in all participants, such short-term axial responses could represent a useful surrogate marker of pharmacologic efficacy. Conversely, 25% of our cohort did not respond to adjunct atropine, showing no reduction in progression rate under combination therapy—consistent with the non-responder subgroup reported by Sim et al.^26^. The predominant shared feature among these patients was parental myopia: four of five had two myopic parents, with extreme (≥–10 D) maternal and moderate (–3 to − 6 D) or high (>–6 D) paternal myopia. These observations align with recent evidence that the magnitude of parental myopia significantly influences a child’s progression risk^29,30^ and that maternal myopia may have a stronger impact^31,32^, particularly among treatment-resistant progressors^6^.

### Pupil dynamics and DIMS treatment response

Our findings are consistent with prior work showing that the pupillary light reflex reflects autonomic nervous system activity and can serve as an indicator of cholinergic–adrenergic balance^13,14,16,17^. Altered pupillary dynamics have been reported in various systemic and neurological conditions^33,34^, and reduced autonomic tone has been associated with greater axial elongation in myopic children^17^. The slower constriction kinetics observed among progressors in our cohort may therefore indicate diminished parasympathetic influence. Similar associations between slower pupillary light reflex responses and higher degrees of myopia have been described elsewhere^17,35^. Notably, pharmacologic studies also report reduced constriction—but not dilation—velocity following low-dose atropine instillation in healthy adults^36^, supporting a possible link between cholinergic modulation and constriction dynamics.

Alternatively, slower pupil constriction could reflect reduced retinal ON-pathway signalling. Poudel and colleagues^37^ proposed that inadequate stimulation of ON pathways under low-light or low-contrast conditions may weaken these circuits and diminish their inhibitory drive on ocular growth. Their subsequent study^16^ showed that greater myopia severity is associated with slower and less responsive ON-pathway activity resulting in less effective pupil constriction. As several studies have linked deficient ON-pathway activation to myopia progression^16,37,38^, it is plausible that slower constriction kinetics in our progressor group reflect reduced ON-pathway efficiency, increasing susceptibility to continued elongation. Extending these findings to an age- and refraction-matched, treatment-resistant cohort, our results suggest that altered pupil constriction dynamics could help identify children who may benefit from earlier escalation to pharmacologic therapy.

### Limitations and future directions

Our study has several limitations that should be considered when interpreting the results. The longitudinal combination-therapy cohort was of moderate size, which may limit statistical power and increase the risk of type II error, although it was sufficient to reveal clinically meaningful changes over one year. Nevertheless, the relatively small sample size restricts the generalizability of our findings and underscores the need for subsequent large-scale, multicenter validation studies to confirm these results. In addition, larger studies make it possible to investigate potential risk factors for inadequate response in combination treatment. Longer-term follow-up will be necessary to determine the durability of these effects. Prospective studies with larger samples are also required to confirm the predictive value of pupillometric parameters. Second, the exclusion of low-quality pupillometry recordings led to groups that were not perfectly matched, although statistical adjustments helped minimize this imbalance. In addition, the combination therapy group did not include a DIMS + placebo control arm; therefore, the potential contribution of placebo effects to the observed changes in myopia progression cannot be entirely excluded. Future randomized controlled trials incorporating appropriate placebo-controlled groups would help to better isolate treatment-specific effects. Finally, while the precise mechanisms underlying altered constriction dynamics remain to be clarified, our findings provide a rationale for future work integrating retinal imaging, choroidal thickness measurements, and dynamic pupillometry to better elucidate the physiological pathways involved.

## Conclusion

In summary, the present work shows that adjunct 0.025% atropine can effectively slow progression in DIMS non-responders and that differences in pupil constriction kinetics may help identify children who require earlier escalation to combination therapy. Together, these results represent a step toward personalized, physiology-guided myopia management informed by objective functional measures. Although the precise mechanisms underlying altered constriction dynamics remain to be clarified, our findings provide a rationale for future studies combining retinal imaging, choroidal monitoring, and dynamic pupillometry to further elucidate the physiological basis of treatment response.

## Data Availability

Data is available upon reasonable request.
